# Chitosan/PAMAM/Hydroxyapatite Engineered Drug Release Hydrogels with Tunable Rheological Properties

**DOI:** 10.3390/polym12040754

**Published:** 2020-03-31

**Authors:** Alessandro Pistone, Daniela Iannazzo, Consuelo Celesti, Cristina Scolaro, Salvatore V. Giofré, Roberto Romeo, Annamaria Visco

**Affiliations:** 1 Department of Engineering, University of Messina, Contrada Di Dio, I-98166 Messina, Italy; ccelesti@unime.it (C.C.); cscolaro@unime.it (C.S.); avisco@unime.it (A.V.); 2 Department of Chemical, Biological, Pharmaceutical and Environmental Sciences, University of Messina, Viale Annunziata, I-98168 Messina, Italy; sgiofre@unime.it (S.V.G.); robromeo@unime.it (R.R.)

**Keywords:** chitosan, tissue engineering, hydroxyapatite, PAMAM dendrimer, rheological properties

## Abstract

In this paper, a new formulation of biodegradable and bioresorbable chitosan-based hydrogel for controlled drug release was investigated. A chitosan–dendrimer–hydroxyapatite hydrogel, obtained by covalently grafting chitosan powder with an hyperbranched PAMAM dendrimer followed by in-situ precipitation of hydroxyapatite and gelification, was synthesized and characterized by FTIR, NMR, TGA, XRD and rheological studies. The hydrogels have been also doped with an anti-inflammatory drug (ketoprofen) in order to investigate their drug release properties. Chemical and chemical-physical characterizations confirmed the successful covalent functionalization of chitosan with PAMAM and the synthesis of nanostructured hydroxyapatite. The developed hydrogel made it possible to obtain an innovative system with tunable rheological and drug-releasing properties relative to the well-known formulation containing chitosan and hydroxyapatite powder. The developed hydrogel showed different rheological and drug-releasing properties of chitosan matrix mixed with hydroxyapatite as a function of dendrimer molecular weight; therefore, the chitosan–dendrimer–hydroxyapatite hydrogel can couple the well-known osteoconductive properties of hydroxyapatite with the drug-release behavior and good processability of chitosan–dendrimer hydrogels, opening new approaches in the field of tissue engineering based on biopolymeric scaffolds.

## 1. Introduction

The development of biomaterials for application in medicine represents a fascinating challenge in the field of materials science. In recent decades, biodegradable polymers have received increasing interest in the field of tissue engineering [1,2]. Compared to other traditional implant materials, such as metals and non-degradable polymers, biodegradable polymers, once implanted in vivo, degrade over time by either enzymatic, microbial, or chemical processes, producing non-toxic byproducts which are then excreted and metabolized harmlessly [3,4,5,6,7,8,9]. Among the different 3D biopolymeric scaffolds, polysaccharide-based hydrogels composed of chitosan, alginate, dextran or hyaluronic acid have been widely investigated for bone and soft tissue engineering applications, owing to their high biocompatibility and biodegradability, low cost, ease of handling, viscoelastic properties comparable to soft tissues, possibility of gelation in situ, and ability to provide an ideal hydrated environment for the growth of cells or tissues [10]. Moreover, based on their chemistry, they can be cross-linked, allowing the incorporation of cells, growth factors and the controlled release of bioactive molecules [11].

Among the different natural polysaccharides, chitosan (CS), obtained from the deacetylation of chitin has been widely investigated for the development of scaffold used to repair or regenerate organ or tissues, due to its good biocompatibility and biodegradability and to the biological properties derived from its chemical composition [12].

The chemical functionalization of chitosan with biocompatible polymeric structures, such as grafting copolymerization or coupling with biodegradable dendrimers and cyclodextrins, has shown to improve relevant features for its use in hydrogel formulations [13]. Dendrimers, highly branched globular macromolecular structures with nanometer-scale dimensions, possess a typical tree-like architecture able to influence the chemical and physical properties of chitosan/dendrimer hybrids, especially at high molecular weights, thus offering several possibilities in biomedical applications [14,15,16]. Among the different types of synthetic dendrimers, the poly(amidoamine) (PAMAM) dendrimers, which consist of alkyl-diamine cores, represent the most common class of biodegradable dendrimers and have been investigated for the targeted delivery of drugs and genes as well as for the development of co-delivery systems [17].

The insertion of nanosized inorganic material such as hydroxyapatite (HA), the main mineral component of bones and teeth, into polysaccharide-based hydrogels have also proved to improve mechanical properties and bioactivity of scaffolds for bone tissue engineering [18,19]. In particular, the addition of HA into chitosan-based hydrogel has shown to provide better cell and protein adhesion, enhanced cell proliferation and higher osteogenic gene expression, relative to chitosan hydrogel alone [20,21]. Moreover, while a burst and fast drug release for pristine chitosan network is often reported, a slower and more controlled drug release is generally observed from chitosan/HA hybrid composites [22,23,24].

On the basis of these considerations, we developed a new formulation for soft tissue engineering applications and for controlled drug release, constituted by a chitosan–dendrimer–hydroxyapatite hydrogel obtained by covalently grafting chitosan powder with hyperbranched PAMAM dendrimers with different molecular weights followed by in-situ precipitation of hydroxyapatite and gelification; in such a manner, the coupling of dendrimeric structure and hydroxyapatite together inside the chitosan matrix made it possible to obtain an innovative system with tunable rheological and drug-releasing properties relative to the well-known formulation containing chitosan and hydroxyapatite powder. Chitosan-based hydrogels were cross-linked using the cross-linking agent genipin, naturally found in *Gardenia jasminoides* fruits, which ensures very low cytotoxicity and comparable mechanical and biodegradable properties relative to other cross-linking agents (like glutaraldehyde) [25,26].

The synthesized hydrogel scaffolds were investigated for their chemical, physical and rheological properties and their drug release abilities were evaluated using ketoprofen as a model drug, which was included in the scaffold during the gelation procedure. The results highlighted that the presence of dendrimeric moieties with different molecular weight can allow different rheological properties of a chitosan matrix mixed with hydroxyapatite; furthermore, the chitosan–dendrimer–hydroxyapatite hydrogel can allow a drug release kinetic similar to the parent hydrogels without dendrimeric moieties with improved amount of drug released.

## 2. Materials and Methods

### 2.1. Materials

The reagents chitosan (medium molecular weight, deacetylation 75%–85%), acetic acid (99.9%), ethylenediamine (99.5%), methyl acrylate (99%), calcium nitrate tetrahydrate (>99%), potassium tert-butoxide (>98%), methanol (99%), tetrahydrofuran (>99%), ammonium phosphate dibasic (>98%), ammonia solution (>99.5%) and ketoprofen (>99%), were purchased from Sigma Aldrich (St. Louis, MO, USA); genipin (>98%) was purchased from Carbosynth (St. Gallen, Switzerland). All reagents and solvents were used without further purification. NMR spectra were recorded on a Varian Unity Inova instrument operating at 500MHz and chemical shifts were reported in ppm (σ) using TMS as internal standard. The infrared spectra were obtained using a Fourier-Transform Infrared (FT-IR) spectrometer VERTEX 80/80v (Bruker Optik GmbH, Ettlingen, Germany), by the method of KBr pellets in the range of 4000–500 cm^−1^. X-ray diffraction (XRD) experiments were performed using a BrukerD8 Advance diffractometer (Bruker, Karlsruhe, Germany) at room temperature with a Bragg–Brentano theta-2theta configuration and Cu Ka radiation (40 V, 40 mA). The XRD patterns were collected in the range 10°–60° with a step of 0.2 °/s. The obtained diffraction peaks were compared with those in the Joint Committee on Powdered Diffraction Standards (JCPDS) database. Thermogravimetric analyses (TGA) were performed using a TAQ500 instrument (TA Instruments, New Castle, DE, USA) from 100 to 700 °C, with a rate of 20 °C per minute, under an air atmosphere. Optical images were recorded by means of a Hirox digital microscope, mod. KH8700 (Hirox, Tokyo, Japan) by mounting a MX(G)-5040Z lens at room temperature. Rheological property measurements were carried out by means of a rotational rheometer (Mod. SR5, Rheometric scientific, Piscataway, NJ, USA) with parallel plate geometry at 37 °C. UV spectra have been performed by a Thermo Nicolet mod., Evolution 500 spectrophotometer, measuring the drug absorbance at 260 nm.

### 2.2. Methods

#### 2.2.1. Synthesis of PAMAM Dendrimers and Chitosan–PAMAM Chains

Chitosan chains functionalized with poly(amidoamine) (PAMAM) dendrimers were synthesized starting from PAMAM-CO_2_Me dendrimer of generation 1.5, 2.5 and 3.5 which were produced as reported in literature by the repetition of two processes: (a) Michael addition of methyl acrylate (MA) to the amino groups of the initiator core ethylenediamine(EDA) and (b) amidation reaction of ester moieties with the amino groups of EDA [27]. Briefly, Michael addition was performed by reaction of EDA (6 g, 0.1 mol) with MA (86 g, 1.0 mol) at 0 °C under vigorous agitation for 10 min. Then, methanol (100 mL) was added, the mixture temperature was allowed to rise to room temperature, and the mixture was left under stirring for 24 h. The solvent and the excess of MA were removed under vacuum at 50 °C, affording the methyl-ester-terminated PAMAM with generation 0.5 (G0.5). Then, EDA (13 g, 0.22 mol) was carefully added to the solution of PAMAM G0.5 (10 g, 0.024 mol in methanol (200 mL) at 0 °C and the mixture was left stirring for 72 h at room temperature. The solvent and the excess EDA were removed under reduced pressure at 70 °C to afford the PAMAM dendrimer G1.0. The Michael and amidification reactions were, step by step, repeated, to obtain the methyl-ester-terminated PAMAM dendrimer with generation G1.5, G2.5 and G3.5. After removal of the solvent and the excess MA, under vacuum at 50 °C, the samples were characterized by FTIR and ^1^H NMR and used for the subsequent conjugation with chitosan without further purification (^1^H NMR (500 MHz, CDCl_3_, ppm) δ H: 3.66 (48H, s), 3.28–3.26 (24H, m), 2.77–2.74 (60H, m) 2.49–2.42 (80H, m)).

Each of the so-formed dendrimers of PAMAM-CO_2_Me was treated with a solution of potassium tert-butoxide (80 mg, 0.71 mmol) in THF (5 mL, containing 0.2% of H_2_O) and was left under stirring in air for 1 min at room temperature. A dispersion of chitosan (1 g) in deionized water (5 mL) was then added to each mixture containing the different generation of dendrimer (0.35 mmol), which was left to stir at room temperature for 2 hrs. Then, after removal of the solvent under vacuum, the mixture was diluted with water and purified using a dialysis bag (MW of 12,000 Da) for 8 h. After removal of water under vacuum at 50 °C, the mixture was dried at 50 °C for 24 h at the vacuum drying pressure of 65 mbar. The sample was characterized by FTIR and ^1^H NMR and used for the subsequent reactions without further purification (^1^H NMR (500 MHz, D2O, ppm) δ H: 5.0 (CS-H1, m), 3.94-3.92 (CS-H2, CS-H6, m), 3.85–3.71 (CS-H3, CS-H4, CS-H4, m), 3.65 (PAMAM, s), 3.26–3.25 (PAMAM, m), 2.76–2.74 (PAMAM, m) 2.53–2.40 (PAMAM, m), 1.85 (CS-C(O)CH3, s)).

#### 2.2.2. Synthesis of Chitosan Hydrogel (CS Sample)

Chitosan powder with medium molecular weight (240 mg) was dispersed in an aqueous solution of 2% acetic acid (20 mL) at 45 °C, for 30 min. Then, the cross-linking agent genipin (24 mg, 0.1 mmol) was slowly added to the mixture. The so-formed hydrogels were rinsed with deionized water and dried at 37 °C for 24 h at the vacuum drying pressure of 65 mbar.

#### 2.2.3. Synthesis of Chitosan-Hydroxyapatite Hydrogel (CS-HA Sample)

Chitosan powder with medium molecular weight (240 mg) was dispersed in an aqueous solution of 2% acetic acid (20 mL) at 45 °C, for 30 min. Then, Ca(NO_3_)_2_·4H_2_O (368 mg, 1.56 mmol) and (NH_4_)_2_HPO_4_ (66 mg, 0.5 mmol) were added to the dispersion under vigorous agitation and the mixture was stirred for 30 min until complete dissolution of calcium and phosphate salts. Subsequently, genipin (24 mg, 0.1 mmol) was added slowly added to the mixture. The mixture was allowed to be gelled for 1 day. The so-formed hydrogel was treated with a 2% NH_3_ solution for 1 h at room temperature to allow the in-situ formation of hydroxyapatite in order to obtain a homogenous distribution of crystalline hydroxyapatite in the biopolymeric matrix [21]. The *CS-HA* hydrogel was rinsed with deionized water until reaching pH 7 and dried at 37 °C for 24 h at the vacuum drying pressure of 65 mbar.

#### 2.2.4. Synthesis of CS-PAMAM-HA Hydrogel (CS-D1.5-HA, CS-D2.5-HA, CS-D3.5-HA Samples)

Each sample of CS-PAMAM (240 mg) dendrimer with generation G1.5, G2.5 and G3.5 was dispersed in an aqueous solution of 2% acetic acid (20 mL) at 45 °C, for 30 min. Then, Ca(NO_3_)_2_·4H_2_O (368 mg, 1.56 mmol) and (NH_4_)_2_HPO_4_ (66 mg, 0.5 mmol) were added to the dispersion under vigorous agitation and the mixture was stirred for 30 min until complete dissolution of calcium and phosphate salts. Subsequently, genipin (24 mg, 0.1 mmol) was added slowly added to the mixture. The mixture was allowed to be gelled for 1 day. The so-formed hydrogel was treated with a 2% NH_3_ solution for 1 h at room temperature to allow the formation of hydroxyapatite and then rinsed with deionized water until reaching pH 7. Each CS-PAMAM-HA hydrogel obtained by using the three different generations of PAMAM dendrimers was dried at 37 °C for 24 h at the vacuum drying pressure of 65 mbar, to obtain the samples CS-D1.5-HA, CS-D2.5-HA, CS-D3.5-HA.

#### 2.2.5. Synthesis of Ketoprofen-Doped Hydrogel (CS-HA-Keto and CS-D1.5-HA-Keto, CS-D2.5-HA-Keto, CS-D3.5-HA-Keto Samples)

Ketoprofen-doped CS-HA and CS-D1.5-HA, CS-D2.5-HA, CS-D3.5-HA hydrogels were also synthesized following the procedure described in Section 2.2.3 and Section 2.2.4, by adding ketoprofen lysine salt (160 mg, 0.67 mmol) dissolved in 2 mL of deionized water to the mixture before the genipin addition step. Ketoprofen-doped hydrogels were rinsed with deionized water until disappearance of the drug in the washing solutions as evaluated by NMR analysis, and the samples were dried at 37 °C for 24 h at the vacuum drying pressure of 65 mbar up to constant weight. The amount of ketoprofen present in the hydrogels was calculated as the difference between the initial amount of drug dispersed in the mixture during the hydrogels formation and the amount of unbound drug present in the filtrate after washing procedures, and this value was also confirmed after drug release studies. The drug concentration was found to be 4 wt%.

#### 2.2.6. Drug Release Studies

The ketoprofen release from the CS-D1.5-HA-Keto, CS-D2.5-HA-Keto and CS-D3.5-HA-Keto samples was compared with that released from the CS-HA-Keto sample; the effect of the presence of the dendrimeric moieties linked to chitosan chains on the drug release ability with respect to a pure chitosan–hydroxyapatite-based system was investigated. Drug release analyses were performed using a phosphate-buffered saline solution (PBS) at pH 7.4 and a temperature of 37 °C, using a dialysis bag diffusion technique. In a typical experiment, 800 mg of sample, sealed in a dialysis bag, was immersed in 40 mL of PBS in a beaker under slow speed magnetic stirring, Then, 3 mL of sample was collected at different times and the amount of ketoprofen released was quantified by UV–Vis absorption spectra by measuring the absorbance at 260 nm relative to a calibration curve recorded under the same conditions. After each measurement, the same aliquot was added back to the release system.

#### 2.2.7. Rheological Studies

Rheological measurements were performed on cylindrical samples with a diameter of 25 mm and a height of 1 mm. The sample holding system was filled with distilled water and was sealed with an insulating cover to prevent drying of samples during measurements [28]. A dynamic stress sweep test (frequency of 1 Hz) was performed within the stress range of 0.5 Pa–1000 Pa, in order to check the linear viscoelastic region (LVR), in which the stored elastic modulus, G′, and the viscous loss modulus, G′′, are independent of the applied shear stress. Frequency sweep tests (in stress control) were carried out in the frequency range 0.01–200 rad/sec at a constant stress (10 Pa). The rheological properties (frequency response of G′ and of the complex viscosity η*) were checked 30 min after the cross-linking beginning (i.e., after 2.50–3.0 h from the color change). Each test was carried out in duplicate.

## 3. Results and Discussion

### 3.1. Synthesis of Chitosan-Based Hydrogels

#### 3.1.1. Synthesis of Chitosan–PAMAM Chains

**Scheme 1:**
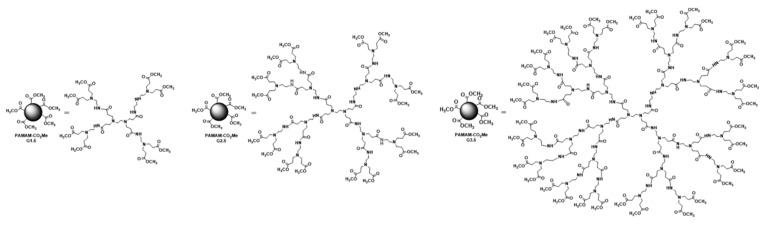

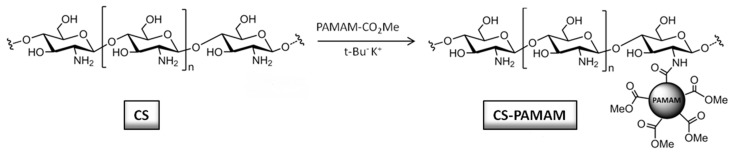
Chemical structure of PAMAM dendrimers and synthesis of CS-PAMAM chains.

The first necessary step for the final development of chitosan–PAMAM based hydrogel concerned the synthesis of chitosan–PAMAM chains. PAMAM dendrimer of generation 1.5, 2.5 and 3.5, containing methyl ester moieties on the external surface, was synthesized according to a reported procedure [27] that consists of two consecutive steps: (a) Michael addition of a primary amino group to methyl acrylate (MA) and (b) amidation reaction of the ester moieties to ethylenediamine (EDA). The tetraester obtained in the first process is called dendrimer of generation 0.5 (G0.5). The repetition of these two processes on PAMAM G0.5 afforded the dendrimer PAMAM G.1.5, and, subsequently, the desired dendrimers PAMAM-CO_2_Me G2.5 and PAMAM-CO_2_Me G3.5, whose structure was confirmed by ^1^H NMR spectroscopy. The NMR analysis of PAMAM-CO_2_Me shows the diagnostic signals of the methyl ester moieties at 3.66 ppm, and three sets of multiplets centered at 3.25, 2.75 and 2.45 ppm, attributable to the methylene protons near the amide groups, the methylene protons of the amine groups and the methylene groups in α position to the ester moieties, respectively. The ester moieties present at the external surface of the home-made dendrimer PAMAM-CO_2_Me were used for the tert-butoxide-assisted amidation reaction with the free nucleophilic amino groups of chitosan (Scheme 1), following a previously reported procedure [29].

The PAMAM-CO_2_Me sample was purified using a dialysis bag able to retain compounds with MW of 12,000 or greater and then the formation of an amide bond between PAMAM dendrimer and chitosan was investigated by ^1^H NMR and FTIR spectroscopy. The ^1^H NMR spectrum of PAMAM-conjugated chitosan (PAMAM-CS sample) shows the diagnostic signal of the proton at C-2 position of the sugar ring, which after amide formation, was shifted from 2.89 for typical chitosan molecule, to 3.94 ppm for the conjugated sample.

**Figure 1:**
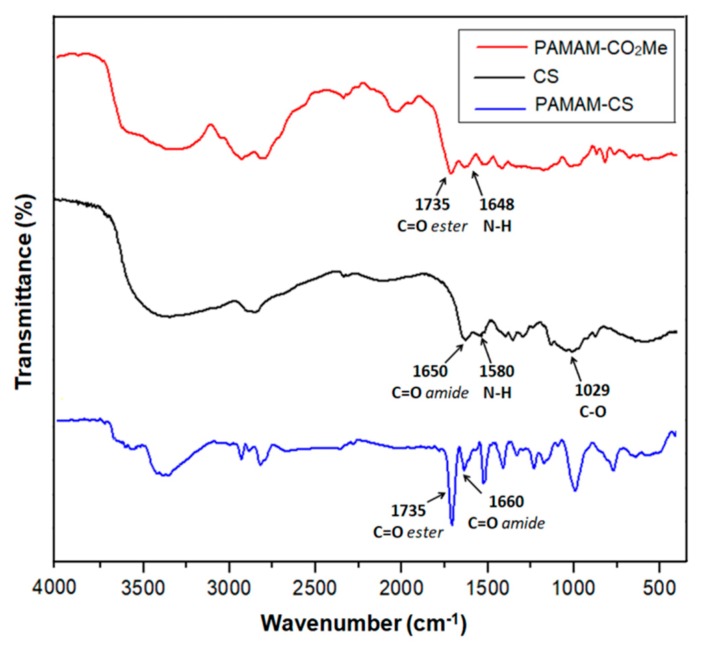
FTIR spectra of CS, CS-PAMAM and PAMAM-CO_2_Me samples.

The FTIR spectroscopy analyses further confirmed the presence of a covalent bond between chitosan and the PAMAM dendrimers (Figure 1). The FTIR spectrum of chitosan shows a band at 1580 cm^−1^ ascribable to N–H bending and a peak at 1029 cm^−1^ corresponding to C–O stretching; moreover, the presence of residual *N*-acetyl groups on the chitosan structure is confirmed by the presence of a peak at 1650 cm^−1^ corresponding to the C=O stretching of the primary amide. The PAMAM-CO_2_Me sample shows peaks at 1735 cm^−1^ and 1648 cm^−1^ corresponding to the C=O stretching of the ester group and to the N–H bending of primary amide, respectively. The conjugated sample PAMAM-CS sample shows the expected presence of to the C=O stretching of the ester group at 1735 cm^−1^ and the additional peak at 1660 cm^−1^ due to the C=O stretching of the new amide bond, thus confirming the success of the amidation reaction between the chitosan and the dendrimer.

#### 3.1.2. Synthesis of CS-D-HA Hydrogels

**Figure 2:**
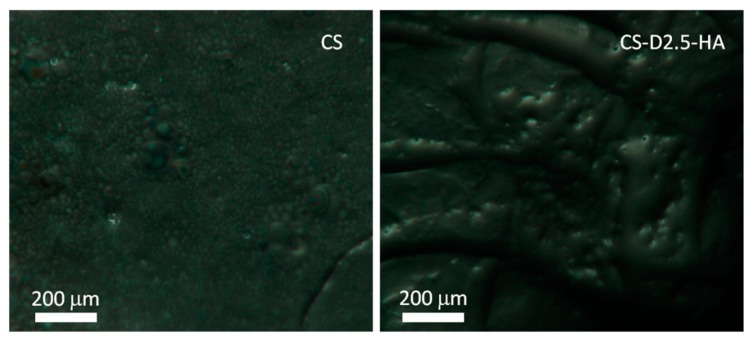
Optical images of CS and CS-D2.5-HA hydrogels.

The functionalization of chitosan chains with dendrimers moieties involves an increase of the surface roughness of the corresponding hydrogels with respect to the parent one obtained with unmodified chitosan (Figure 2).

**Figure 3:**
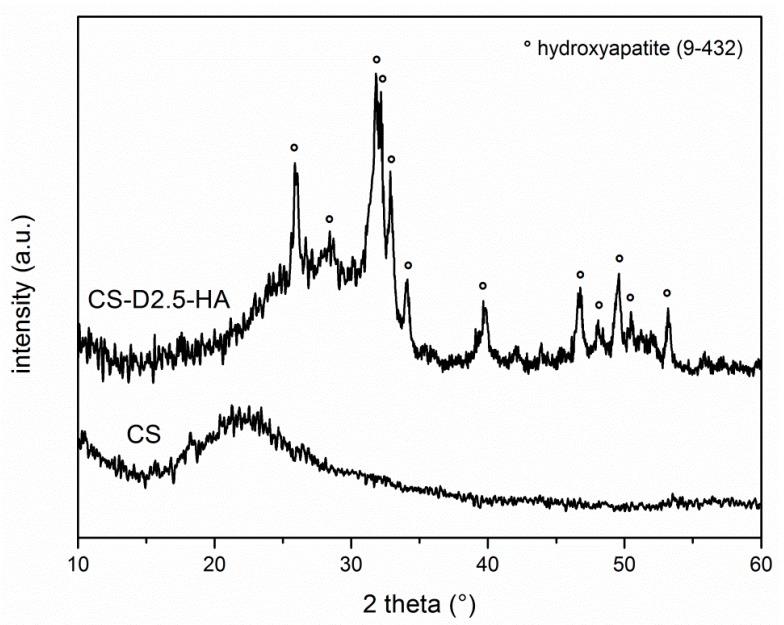
XRD spectra of CS-D-HA hydrogel.

Hydroxyapatite was synthesized inside the hydrogel structure, using an in-situ precipitation procedure which, as reported in the literature, allows a superior homogeneous dispersion of hydroxyapatite with respect to other techniques starting from hydroxyapatite in powder form. XRD analyses (Figure 3) confirm the formation of crystalline hydroxyapatite obtaining a good overlap between the synthesized material and the characteristic line of hydroxyapatite (Ca_10_(PO_4_)_6_(OH)_2_, JCPDS 9–432); in addition, Scherrer’s equation formulation, performed using the (002) reflection peak at 2theta 26° (Equation (1)), showed that the in-situ precipitation makes it possible to synthesize hydroxyapatite with nanosized crystallites in the range of 30 nm, ensuring a high bioactivity behavior because of the faster bioresorption of hydroxyapatite crystallites with surrounding hard tissues [30,31].

(1) $$L=\left(0.9\cdot \lambda \right)/\left(\beta_{002}\cdot \cos\theta \right)$$

where *L* represents the average crystallite size of the hydroxyapatite, *β*_002_ is the peak width at the half maximum expressed in radians, *λ* is the wavelength of the X-ray radiation (Cu Kα radiation, λ = 0.15418 nm) and *θ* (radians) is the (002) peak angular position.

TGA analyses were performed on the CS-D-HA hydrogels and thermal behaviors were compared to CS and CS-HA systems; thermal decomposition of chitosan occurs in several stages comprising dehydration, changes in conformation of molecules, defragmentation and thermo-oxidation, and, so, changes in thermal behavior also reflect structural modifications in the chitosan network.

**Figure 4:**
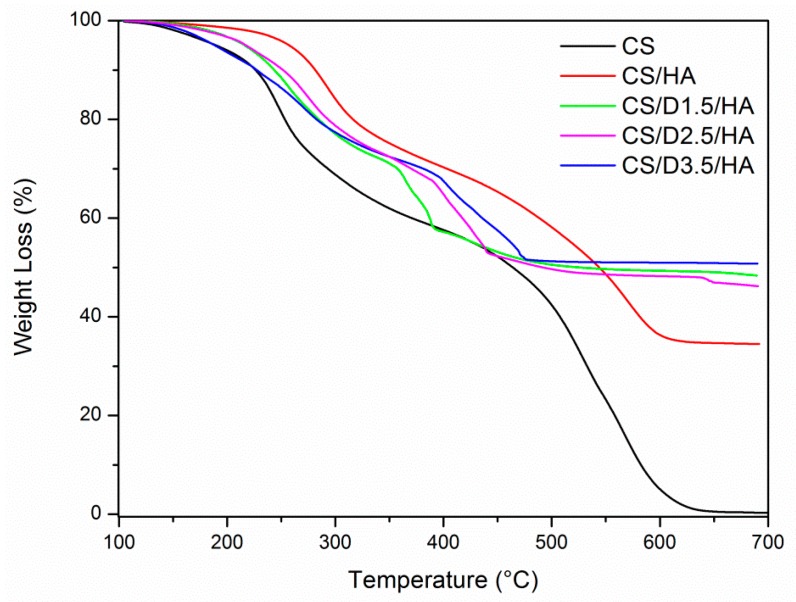
TGA curves for CS, CS-HA and CS-D-HA hydrogels. All experiments were performed under air atmosphere.

All the investigated samples, after a drying procedure under vacuum, were pre-treated at the temperature of 100 °C until a constant weight was achieved and then heated up to 700 °C under air flow, with a rate of 20 °C/min (Figure 4). The chitosan sample (CS hydrogel) was totally oxidized and shows two important weight losses in the range 200–350 °C and 400–600 °C. The hydroxyapatite in the chitosan matrix (CS-HA hydrogel) shifts the degradation step towards higher temperatures confirming, as reported in literature, the hindering effect of hydroxyapatite in thermal oxidation of polymeric matrix [32]; the residual weight of 35 wt% corresponds to the hydroxyapatite loading in the hydrogel. For the hydrogels obtained with chitosan–PAMAM chains (CS-D1.5-HA, CS-D2.5-HA and CS-D3.5-HA samples) it is possible to highlight two regions where the mass loss occurs. In the temperature range of 200–350 °C, the mass loss proceeds with a similar profile of CS and CS-HA samples due to the initial thermodegradation of chitosan matrix; a sharp mass loss was then observed at increasing temperature ranges for CS-D1.5-HA (350 °C < *T* < 400 °C), CS-D2.5-HA (385 °C < *T* < 450 °C) and CS-D3.5-HA (400 °C < *T* < 500 °C) samples respectively; the dendrimeric structure in the chitosan chains lowers the thermal oxidation resistance of hydrogels promoted by hydroxyapatite, since the presence of hyperbranched PAMAM dendrimers hinders the close packing between chitosan chains, hence increasing the biopolymer chain spacing, which, in turn, increases the chain mobility and chain scission. A residual weight of about 50 wt% of hydroxyapatite was observed for all the chitosan–PAMAM–hydroxyapatite-based hydrogels, indicating that the spacer effect of dendrimeric moieties makes it possible to incorporate a greater amount of hydroxyapatite inside the network of chitosan chains.

### 3.2. Drug Release Studies

**Figure 5:**
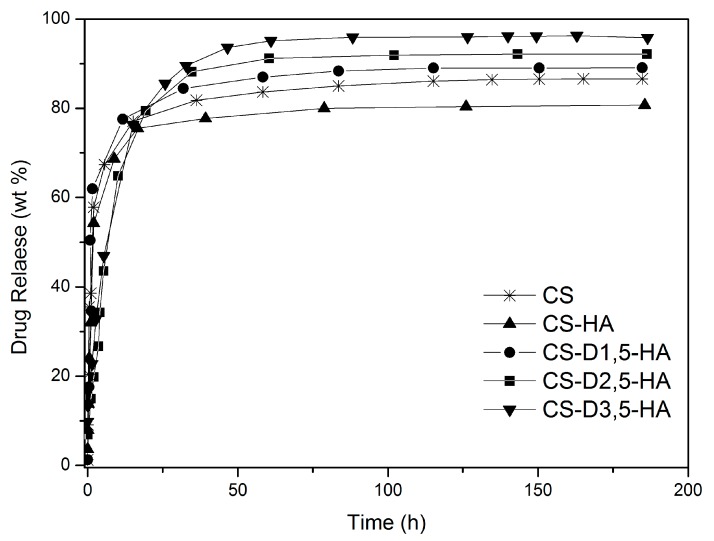
Ketoprofen release from CS-HA- and CS-D-HA-based hydrogel in PBS (pH 7.4), at 37 °C.

In order to investigate the effect of the dendrimeric moieties on the drug release behavior, the hydrogels were loaded with an aqueous solution of ketoprofen (160 mg/2 mL) at the concentration of 4% weight with respect to the weight of samples, and their release properties were compared. The drug release was investigated at the temperature of 37 °C, in buffer phosphate solution (pH = 7.4) and using the dialysis bag diffusion technique in an orbital shaker rotating at 30 rpm. The amount of drug released was quantified by UV-Vis absorption spectra, by measuring the drug absorbance at 260 nm (Figure 5). All the samples showed similar drug release profiles within the first 16 h, despite the presence of crystalline hydroxyapatite; this behavior could be associated with the chitosan matrix that rules the drug release outside the hydrogel. After this first step, the cumulative values of ketoprofen released were found in the range from 78 to 95 wt%. The hydrogels based on chitosan–PAMAM chains showed a higher amount of ketoprofene released than the hydrogels based on unmodified chitosan due to the spacer effect of dendrimeric structures among the chitosan chains.

### 3.3. Rheological Studies

**Figure 6:**
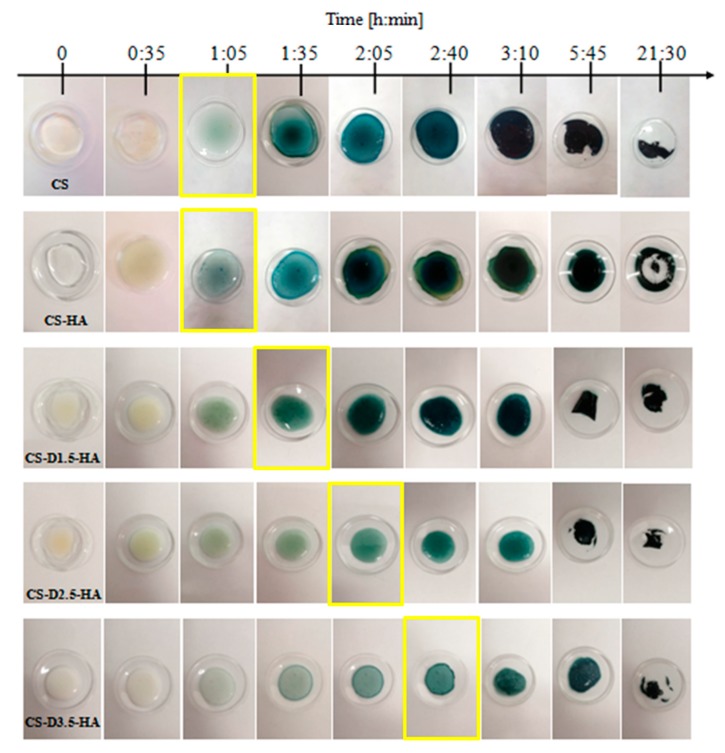
Photographs of the chitosan-based hydrogels collected during their cure reaction at 37 °C.

In this study, hydrogels were formed using the natural and biocompatible cross-linking agent genipin. Since this molecule can cross-link macromolecules by binding two amine groups between neighboring chains, the degree of cross-linking and the gelation time mainly depends on the percentage of amine groups that are accessible to this molecule. As is known, the cure reaction evolves through three physical states: liquid, gel (or gelling) and solid (or vitrification). All the samples, transparent at the beginning, change color during the cure reaction, to light blue (when gelling starts) and then black (when the cross-linking reaction is completed, vitrification). Thus, since the cure reaction of all the samples evolves with the same above-mentioned chromatic variation, pictures of the samples captured at different times can be used to follow the effect of the different chemical structures of chitosan-based systems on the cross-linking rate. Figure 6 shows the cure reaction development at 37 °C of the CS, CS-HA, CS-D1.5-HA, CS-D2.5-HA, CS-D3.5-HA samples deposited in a glass crucible.

The starting gelling time noticed from the pictures of the samples is listed in Table 1; it is defined by the change of color, from transparent to light blue, shown in the pictures of Figure 6. The pure chitosan begins to gel at 1 h and 35 min; the addition of ionic precursor of hydroxyapatite promotes the cross-linking reaction so that chitosan chains start to gelling at lower times (1 h and 5 min). The presence of dendrimeric moieties bonded to chitosan chains shifts the beginning of gelling reaction toward higher times with the increase of the dendrimer size, from about 1 h and 35 min for CS-D1.5-HA sample to 2 h and 40 min for CS-D3.5-HA one.

**Figure 7:**
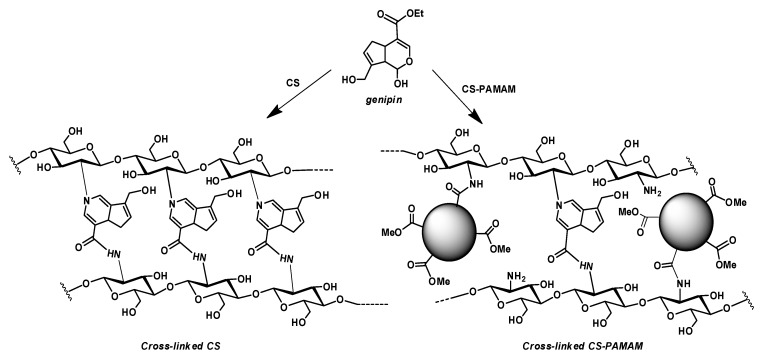
Hindering effect of dendrimeric presence towards cross-linking efficiency of chitosan chains.

Due to the steric hindrance caused by dendrimeric PAMAM macromolecules bonded to chitosan chains, the CS-D-HA hydrogels showed a more delayed gelation time with respect to the hydrogel obtained from unmodified chitosan (CS and CS-HA samples), hindering and therefore delaying the natural development of the polymer cross-linking reaction. (Figure 7).

We investigated the rheological properties of the synthesized hydrogels, carrying out rheological property measurements in dynamic stress sweep and frequency sweep mode in order to check the linear viscoelastic region (LVR) and to calculate the main rheological properties (i.e., frequency response of G′ and of the complex viscosity η*). The value of 10 Pa was found to be valid for our samples, within the LVR, according to literature [33,34]

Both G’ and η* values of all the samples grow, at parity of frequency, in the order:

CS-D3.5-HA > CS-D2.5-HA > CS-D1.5-HA > CS-HA > CS

**Figure 8:**
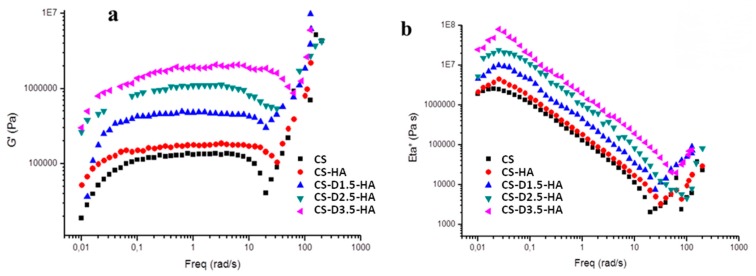
Elastic G’ modulus (**a**) and complex viscosity (Eta*) (**b**) vs. frequency for CS, CS-HA, CS-D1.5-HA, CS-D2.5-HA and CS-D3.5-HA samples.

This trend is always the same, both at a low frequency of 0.1 rad/sec, as well as at a higher frequency of 10 rad/sec (Figure 8). As an example, G’ at a frequency of 0.1 rad/sec grows from 112752 Pa for the CS sample to 1,36962E6 Pa for the CS-D3.5-HA sample. Similarly, at a frequency of 10 rad/sec it grows from 114072 Pa for CS sample to 1,8724E6 Pa for the CS-D3.5-HA sample. This trend is repeated in the η* values (see details in Table 1).

Chitosan has a soft consistency and a ductile behavior [34]. Rheological test results suggest that the addition of HA to the chitosan improves the stiffness and, hence, the viscosity of this polymer. Thus, the CS-HA material has a lower ductility than that of pure CS. The presence of dendrimers of increasing size in the CS-D-HA hydrogels further changes the rheological response, showing an increase of stiffness and viscosity as a result of the lowering of reciprocal movement of biopolymeric chains due to the steric hindrance of the dendrimer.

## 4. Conclusions

A new generation of chitosan-based hydrogels was successfully synthesized, coupling the biopolymeric matrix with hyperbranched PAMAM dendrimer moieties with different sizes and well-dispersed hydroxyapatite, and characterized by NMR, FTIR; XRD, TGA, optical microscopy and rheological studies. Hydrogels were also loaded with ketoprofen, as a drug model, in order to investigate the drug release behavior.

Hydroxyapatite was precipitated inside the biopolymeric matrix, obtaining a nanocrystalline structure, while dendrimeric units with different sizes covalently bonded to chitosan can allow different rheological properties of the chitosan matrix mixed with hydroxyapatite; furthermore, the chitosan–dendrimer–hydroxyapatite hydrogel can allow a drug release kinetic similar to the parent hydrogels without dendrimeric moieties, with an improved amount of drug released. The chitosan–dendrimer–hydroxyapatite hydrogel can couple the well-known osteoconductive properties of hydroxyapatite with the drug release behavior and good processability of chitosan–dendrimer hydrogels, opening new approaches in the field of tissue engineering based on biopolymeric scaffolds.

## Figures and Tables

**Table 1:** Sample code, composition and rheological features.

Sample Code	Sample Composition	Gelling Time (Figure 6) (h:m)	Freq. 0.1 rad/s		Freq. 10 rad/s	
			G’ (Pa)	η* (10^6^ Pa∙s)	G’ (Pa)	η* (10^6^ Pa∙s)
**CS**	**100 wt% pure chitosan**	1:05	112,752	1.128	114,072	0.011409
**CS-HA**	65 wt% pure chitosan + 35 wt% hydroxyapatite	1:05	151,501	1.519	171,425	0.017144
**CS-D1.5-HA**	50 wt% dendrimer modified chitosan + 50 wt% hydroxyapatite	1:35	424,121	4.401	428347.7	0.033921
**CS-D2.5-HA**	50 wt% dendrimer modified chitosan + 50 wt% hydroxyapatite	2:05	838,935	10.665	808,642	0.081778
**CS-D3.5-HA**	50 wt% dendrimer modified chitosan + 50 wt% hydroxyapatite	2:40	1.36962 × 10^6^	18.821	1.8724 × 10^6^	0.191665
